# Assessment of gut microbiota populations in lean and obese Zucker rats

**DOI:** 10.1371/journal.pone.0181451

**Published:** 2017-07-13

**Authors:** Reza Hakkak, Soheila Korourian, Steven L. Foley, Bruce D. Erickson

**Affiliations:** 1 Department of Dietetics and Nutrition, University of Arkansas for Medical Sciences, Little Rock, Arkansas, United States of America; 2 Department of Pediatrics, University of Arkansas for Medical Sciences, Little Rock, Arkansas, United States of America; 3 Arkansas Children’s Research Institute, Little Rock, Arkansas, United States of America; 4 Department of Pathology, University of Arkansas for Medical Sciences, Little Rock, Arkansas, United States of America; 5 National Center for Toxicological Research, US Food and Drug Administration, Jefferson, Arkansas, United States of America; University of North Carolina at Chapel Hill, UNITED STATES

## Abstract

Obesity has been on the rise in the US and worldwide for the last several decades. Obesity has been associated with chronic disease development, such as certain types of cancer, type 2 diabetes, cardiovascular disease, and liver diseases. Previously, we reported that obesity promotes DMBA-induced mammary tumor development using the obese Zucker rat model. The intestinal microbiota is composed of a diverse population of obligate and facultative anaerobic microorganisms, and these organisms carry out a broad range of metabolic activities. Obesity has been linked to changes in the intestinal microbiota, but the composition of the bacterial populations in lean and obese Zucker rats has not been carefully studied. Therefore, the objective of this study was to determine the effects of obesity on the gut microbiota in this model. Lean and obese female Zucker rats (n = 16) were fed an AIN-93G-like diet for 8 weeks. Rats were weighed twice weekly, and fecal samples were collected at the beginning and end of the experiment. 16S rRNA gene sequencing was used to evaluate the composition of the fecal bacterial populations. At the outset of the study, the lean rats exhibited much lower ratios of the Firmicutes to Bacteroidetes phyla than the obese rats, but after 60 days, this ratio in the lean rats exceeded that of the obese. This shift was associated with reductions in the Bacteroidaceae, S24-7 and Paraprevotellaceae families in the lean rats. Obese rats also showed increased levels of the genus *Akkermansia* at day 60. PCoA plots of beta diversity showed clustering of the different test groups, indicating clear differences in intestinal microbiota populations associated with both the time point of the study and the lean or obese status in the Zucker rat model for obesity.

## Introduction

Obesity has been epidemic in the US for more than three decades, and the proportion of overweight and obese adults in the population continues to grow. According to a recent data survey of adults in the US, the age-adjusted prevalence of obesity from 2013 to 2014 was 35.0% among men and 40.4% among women [1]. Similarly, many other countries are experiencing dramatic increases in obesity. Worldwide, greater than 1.9 billion adults are overweight—of which 600 million are obese [2]. These statistics have serious health consequences due the association between obesity and the risk for chronic diseases including certain types of cancer, type 2 diabetes, cardiovascular disease, and liver disease [3,4]. The obese or lean state has also been associated with differences in the composition of the intestinal microbiota [5].

The intestinal microbiota is composed of a diverse population of obligate and facultative anaerobic microorganisms that present a broad range of metabolic activities. These organisms typically exist in a symbiotic relationship with the host animal and play important roles in the digestion of dietary components and the metabolism of drugs, xenobiotic compounds, and nutrients [5–7]. The microbiota provides compounds such as short-chain fatty acids, vitamins, and other essential nutrients that are later absorbed by the host, and it also serves as a barrier against colonization of the gastrointestinal tract by pathogenic bacteria. The intestinal microbiota also plays important roles in mucosal and systemic immunity [8,9,10] and can affect energy metabolism and insulin sensitivity in human subjects with metabolic syndrome [11]. The specific population of organisms that comprise the intestinal microbiota in an individual is relatively stable under normal conditions, but changes in diet can cause changes in the distribution of the different bacterial groups [12]. These population changes can affect the metabolic capabilities of the total microbiota population, which can then affect the health of the host [13].

The relationship between obesity and the composition and metabolic activity of the intestinal microbiota is a topic of intense interest in the scientific and medical communities [13,14,15]. Multiple studies have identified specific differences between the microbiota populations in lean and obese subjects [16]. Mice homozygous for the leptin mutation that results in the development of obesity show a reduction in the percentage of Bacteroidetes and an increase in Firmicutes in the microbiota population compared to their wild type siblings when fed the same diet [5]. This effect is not limited to animals with a genetic predisposition to obesity. Diet-induced obesity is also linked to changes in the intestinal microbiota in mice [17]. This connection between adiposity and the gut microbial ecology appears to apply to humans as well [7]. Previously, we reported that obesity promotes DMBA-induced mammary tumor development using the obese Zucker rat model [18]. While obesity has been linked to changes in the intestinal microbiota [5], the composition of the bacterial population in lean and obese Zucker rats has not been carefully studied. Therefore, the main objective of this study was to determine the composition of the intestinal microbiota populations in the lean and obese Zucker rats, and provide a framework for future studies using this animal model into how differences in these populations may influence the development of a wide range of diseases and metabolic conditions.

## Materials and methods

### Experimental design

The animal protocols were approved by the Institutional Animal Care and Use Committee (IACUC) at the University of Arkansas for Medical Sciences. Sixteen 5-week-old female Zucker rats (8 obese fa/fa and 8 lean) were purchased from Harlan Industries (Indianapolis, IN). The rats were genotypically identified fa/fa and lean/lean rats at age of 24 days. Upon receipt, the rats were housed 1 per cage with *ad libitum* access to water and a semi-purified diet similar to AIN-93G (soy oil was replaced with corn oil) (Harlan Teklad; Madison, WI). Following a one-week acclimation period, fecal samples were collected from each rat (age 42 days) and stored at −80°C (day 0). Rats had access *ad libitum* to water and food for the subsequent 8 weeks of the study. Rats were weighed twice weekly, and fecal samples were collected at the beginning and end of the study and stored at −80°C prior to analyses. All rats were euthanized at end of the study in a CO_2_ overdose inhalation chamber followed by decapitation. Statistical analyses on weight gain differences were performed as previously reported [18]. The t-test was used with a significance level of 0.05.

### Fecal microbiome analyses

For analysis of the bacterial population present in the gut, fecal samples were collected from individual animals at the beginning (day 0) and end (day 60) of the experiment. Total microbial DNA was extracted from the fecal samples using the PowerSoil® DNA isolation kit according to the manufacturer’s protocols (Mo Bio Laboratories, QIAGEN; Carlsbad, CA). The isolated DNA served as template for PCR originating from DNA barcoded universal 16S rRNA gene primers that amplified the V3 and V4 variable regions of the 16S rRNA gene [19] (S1 Table).

The sample-specific barcoded PCR products were quantified and the reactions were pooled in equimolar amounts, labeled with the Illumina Nextera XT Library Preparation kit (Illumina, Inc.; San Diego, CA) and sequenced on an Illumina MiSeq instrument with 2 x 300 paired-end read sequencing at the Sequencing Core Facility at the University of Arkansas for Medical Sciences.

The resultant DNA sequencing reads were quality filtered and analyzed using the Quantitative Insights Into Microbial Ecology (QIIME) bioinformatics pipeline [20]. The paired-end sequences were joined using PANDAseq [21] with a quality threshold of 0.9 and sorted based on their source-specific barcodes, and the taxonomic identifications were done through comparison to the GreenGenes database to assign the sequencing reads to the respective specific operational taxonomic units (OTU) for each fecal sample [22,23]. The resultant taxonomic data (numbers of sequence reads per OTU) were exported to Microsoft Excel for further analyses, including graphing of the relative percentages of the different taxa present in each sample and comparison between individual samples and composites of the different sample types (age and obesity status). Statistically significant differences in bacterial groups were identified by analysis of the QIIME biom table using LEfSe [24]. Sample populations were further evaluated in QIIME, including the calculation of Principal Coordinate Analysis (PCoA) plots to compare sample-to-sample population differences as well as rarefaction curves and alpha diversity metrics (Shannon, Chao1, Observed_OTUs, PD_Whole_Tree) to evaluate the within-sample microbial populations. To avoid potential bias due to different sequence coverage for each sample, the data were rarefied to 124,000 sequences per sample prior to PCoA and alpha diversity analyses. The sequences were deposited in the NCBI Sequence Read Archive under study number SRP102313 and accession numbers SRR5366744 –SRR5366767.

## Results

### Body weight

**Fig 1:**
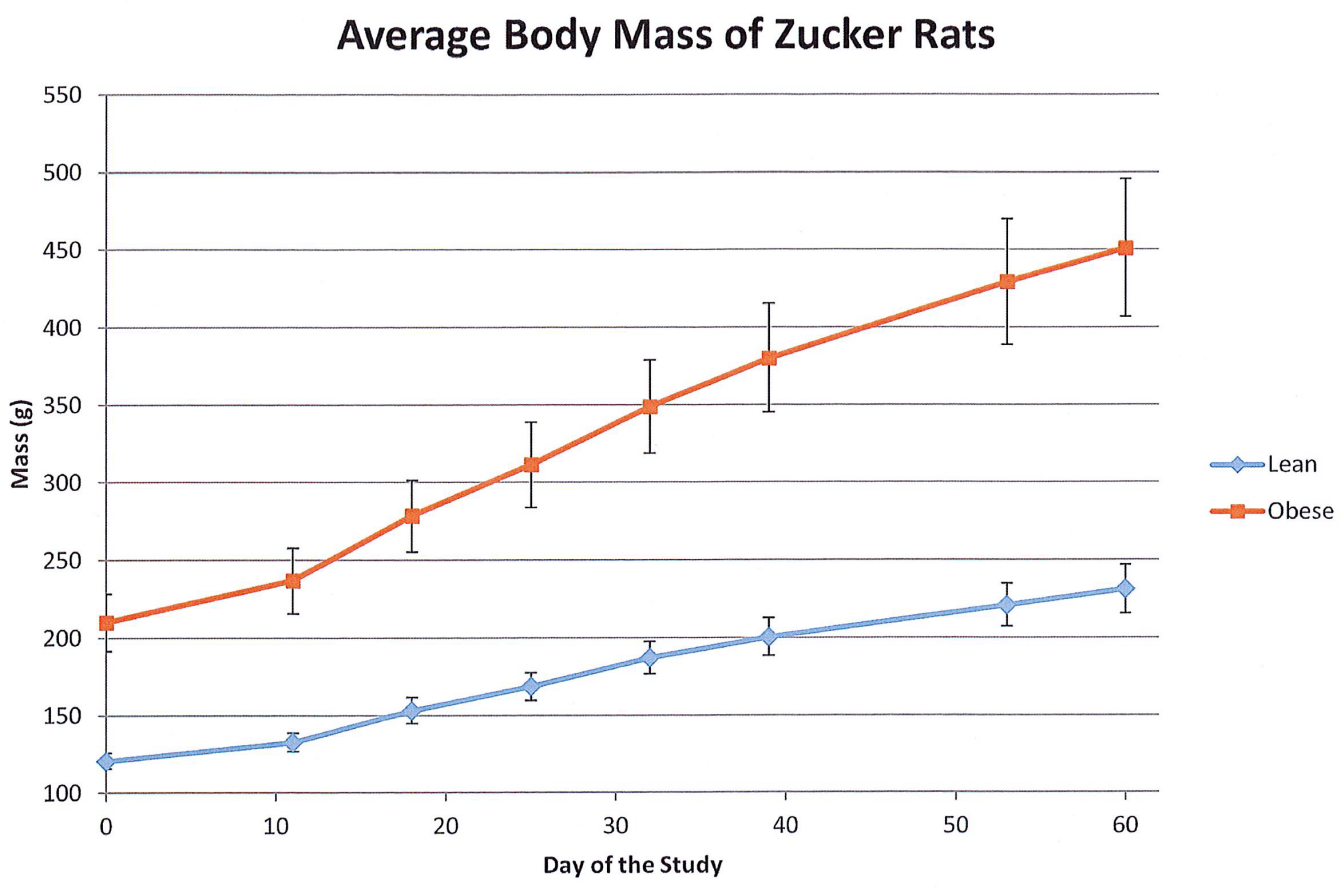
Average body mass of Zucker rats. This graph displays the average body mass for the lean and obese Zucker rats over the course of the 60 day study. Error bars represent ±1Standard Deviation of the mean for the eight rats in each sample group.

All rats gained weight during the course of the experiment. The average body weights at several points are shown in Fig 1. The obese rats had a significantly greater increase in body weight over 60 days of feeding compared to lean rats. At the end of the experiment, mean ± SD body weights for the lean rats were 231 ± 16 g and 451 ± 44 g for obese rats (p<0.001).

### Fecal microbiome analyses

Fecal samples collected at day 0 and day 60 after the one week acclimation period were evaluated using 16S rRNA gene sequencing to determine the composition of the fecal bacterial populations in lean and obese Zucker rats. Illumina sequencing was performed on the V3-V4 region from individual rats at day 0 and day 60. The rats were designated as members of the lean day 0 (Le-0), lean day 60 (Le-60), obese day 0 (Ob-0), or obese day 60 (Ob-60) groups. For the individual fecal samples, the numbers of quality-filtered sequencing reads averaged 291,531 per sample and ranged from 124,247 to 441,653 (Table 1). The individual rarefaction curves are presented in S1 Fig, and the leveling out of the curves demonstrated that the read coverage was likely to capture most of the sample diversity.

**Table 1:** Sequence counts and alpha diversity metrics for individual rat fecal samples.

Sample ID	Sequence Count	Shannon Index	chao1	observed_otus	PD_whole_tree
Le-0-1	227478	8.0111	30286	10240	444.72
Le-0-2	416135	6.7706	14324	4815	255.27
Le-0-3	381662	6.9300	14654	4830	258.01
Le-0-4	395167	6.7651	17287	5511	260.29
Le-60-1	142378	6.7753	13943	4707	253.24
Le-60-2	315142	6.0981	13169	4639	247.03
Le-60-3	287832	6.4943	12927	4414	215.71
Le-60-4	296463	5.8873	9364	3148	176.84
Le-60-5	303116	5.7702	10530	3616	196.63
Le-60-6	147232	6.7569	15066	5405	255.12
Le-60-7	363324	6.4686	11935	4124	221.76
Le-60-8	153850	6.3765	15287	5527	263.34
Ob-0-10	288530	6.7017	11598	4009	216.02
Ob-0-11	283709	6.4723	11139	3701	199.42
Ob-0-12	278800	5.8236	9841	3336	197.76
Ob-0-14	124247	7.1028	14388	4755	249.69
Ob-60-10	224301	6.3432	11300	3995	216.85
Ob-60-11	348406	5.7383	10754	3767	206.71
Ob-60-12	367641	5.8217	11740	4297	219.22
Ob-60-13	378750	6.3935	12921	4354	236.90
Ob-60-14	305834	6.7313	13744	4393	233.53
Ob-60-15	441653	6.7148	14925	5124	264.37
Ob-60-16	280626	6.6711	16416	6114	305.39
Ob-60-9	244461	6.7287	13560	4759	247.11

Sample ID is formatted as [Lean or Obese]-[day of sample collection]-[individual rat number]. Alpha diversity metrics were generated using QIIME, and represent the average from 10 rarefactions of the data to 124,000 sequences per sample.

**Fig 2:**
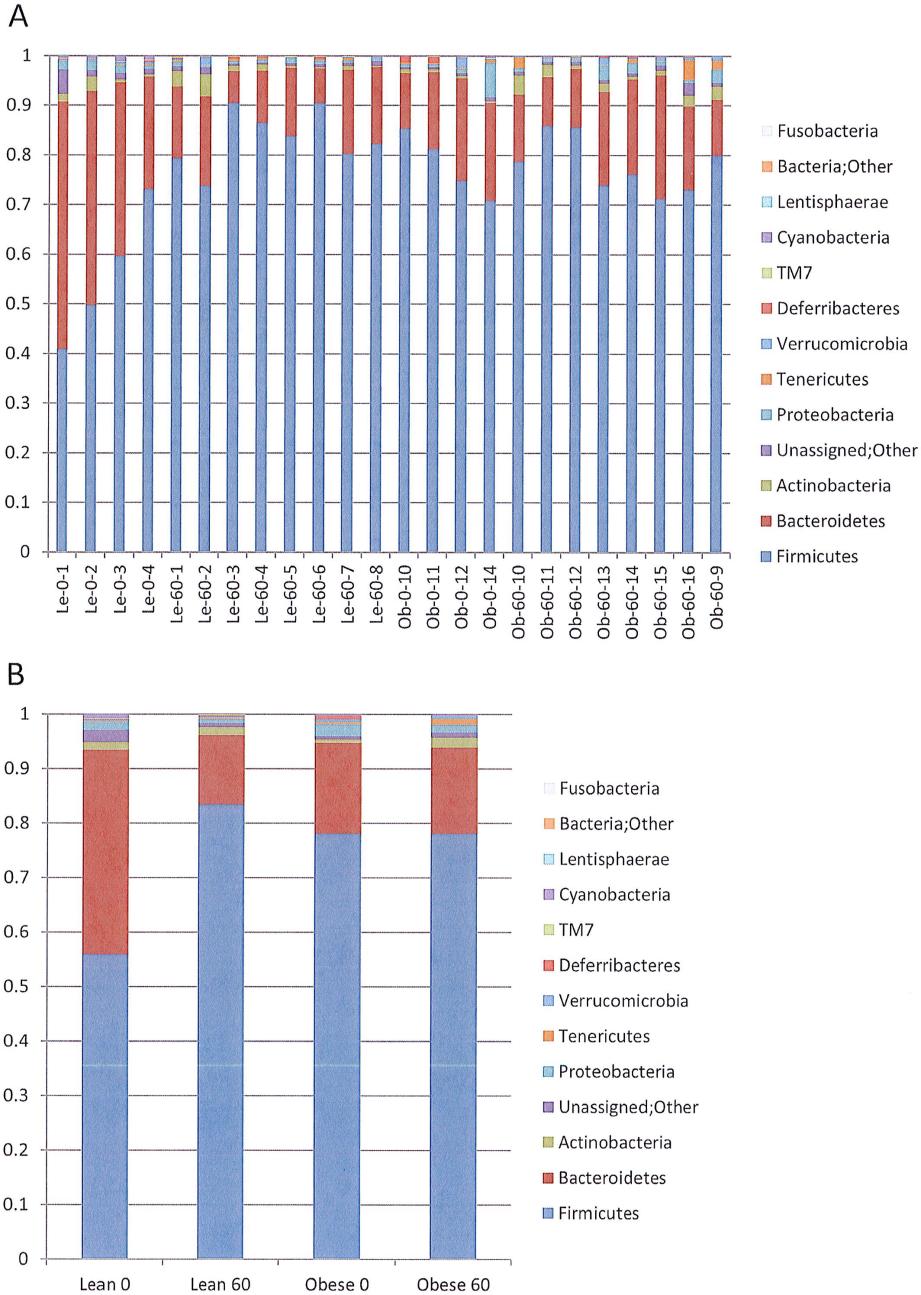
The distribution of bacterial phyla that comprise the intestinal microbiota in the lean and obese Zucker rats. Colored bars represent the relative proportions of bacterial phyla as determined by Illumina sequencing and analysis using QIIME. Panel **A** shows the phylum distribution in the individual rats. Samples designations are Lean or Obese strain (Le or Ob, respectively)–Day of the study (0 or 60)–Individual rat number (1–16). Panel B displays the averaged bacterial phyla within each of the sample groups (Lean day 0, Obese day 0, Lean day 60, and Obese day 60).

Distinct differences in bacterial diversity were observed in the gut microbiota of rats at day 0 and day 60 and to a lesser extent between lean and obese rats at day 60. Fig 2A shows the relative proportions of Phyla for each of the samples evaluated, and Fig 2B shows the composite based on the cumulative findings for the rats in each group. The average ratio of Firmicutes/Bacteroidetes was much lower (1.48) in the feces from lean rats day 0 compared to day 60 (6.51) while these ratios were much closer in the obese rats (4.65 on day 0 and 4.93 on day 60). The obese rats at day 0 had a much higher ratio of Bacteroidetes and Firmicutes to Actinobacteria (31.64 and 147.23) compared to the rats at day 60 (8.51 and 41.96, respectively).

While comprising a small percentage of the total bacterial sequences present in the fecal samples, the Proteobacteria, Tenericutes, and Verrucomicrobia phyla were present in greater numbers in the obese samples than in the lean. At the Family level, the average percentages of Ruminococcaceae were greater in the 60 day samples (Le-60: 29.62 and Ob-60: 28.75%) compared to the day 0 rats (Le-0: 14.91% and Ob-0: 22.86% (S2 Table). The Bacteroidetes present in the fecal samples fell almost exclusively in the order Bacteroidales, with 37.63% of the total sequences in the Le-0 samples, and 16.77%, 12.81%, and 15.81% in the Ob-0, Le-60, and Ob-60 samples, respectively (S2 Table). In the obese samples, the predominant Bacteroidales families (Bacteroidaceae and S24-7) showed little change between day 0 and day 60 (5% and 11% reductions, respectively) while the lean samples showed reductions in the Bacteroidaceae, S24-7, and Paraprevotellaceae families of 42%, 71%, and 95%, respectively, by day 60 (S3 Table).

**Fig 3:**
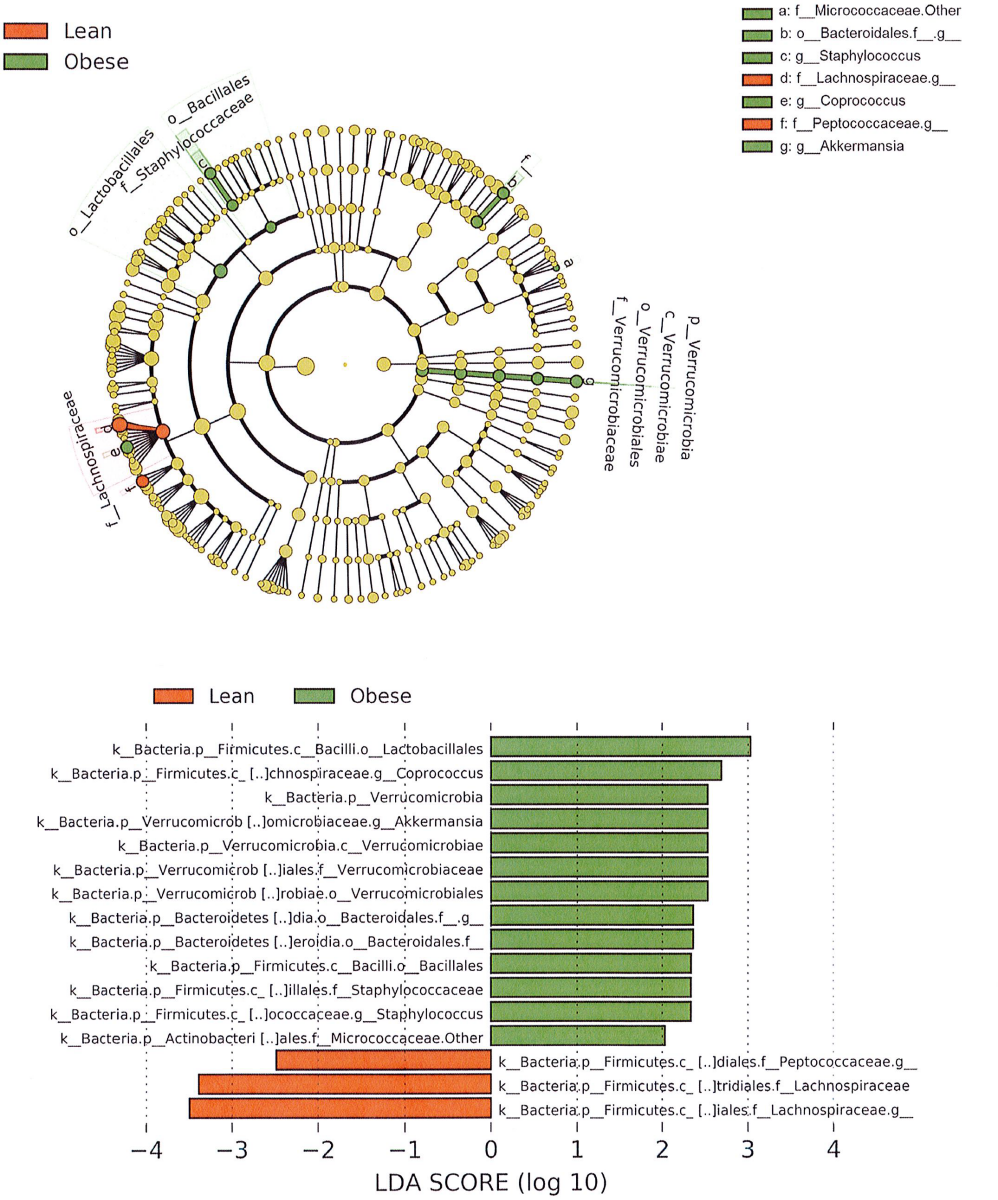
LEfSe analysis identifying statistically significant differences in intestinal microbiota constituents of lean and obese Zucker rats on day 60. The histogram shows the Linear Discriminant Analysis (LDA) scores for bacterial classifications that are significantly elevated in either the Lean (red) or Obese (green) groups. Bars are labeled with the most stringent bacterial classification. The circular cladogram displays the phylogenetic relationship of the bacterial classifications determined to be statistically distinct between the lean and obese groups.

There are several bacterial groups that stand out as present in significantly different proportions in the lean and obese Zucker rats at 60 days–as identified by linear discriminant analysis (Fig 3). The obese rats show higher levels of organisms in the orders Lactobacillales and Bacillales. The increased numbers of Bacillales are due primarily to increased levels of bacteria in the family Staphylococcaceae and genus *Staphylococcus*. Other family-level increases include the family Bacteroidales.f_ (primarily from the genus Bacteroidales.f_.g_) and the family designated as Micrococcaceae.Other. Obese rats also show a significantly higher proportion of the phylum Verrucomicrobia. This difference is due exclusively to elevated levels of the genus *Akkermansia*. While QIIME analysis using the Greengenes database does not identify OTUs to the species level, additional analysis of these *Akkermansia* sequences identified them as from the species *Akkermansia mucinophila*.

Fewer bacterial groups were identified as elevated in the lean Zucker rats. While the family Lachnospiraceae showed elevated numbers in the lean rats, this was due to higher levels of the genus designated Lachnospiraceae.g_. However, the genus *Coprococcus*, also from the family Lachnospiraceae, was present in greater proportion in the obese rats. Lean rats also showed significantly greater numbers of the genus Peptococcaceae.g_. A four-group LDA at days 0 and 60 for both lean and obese Zucker rats is shown in the S2 Fig.

**Fig 4:**
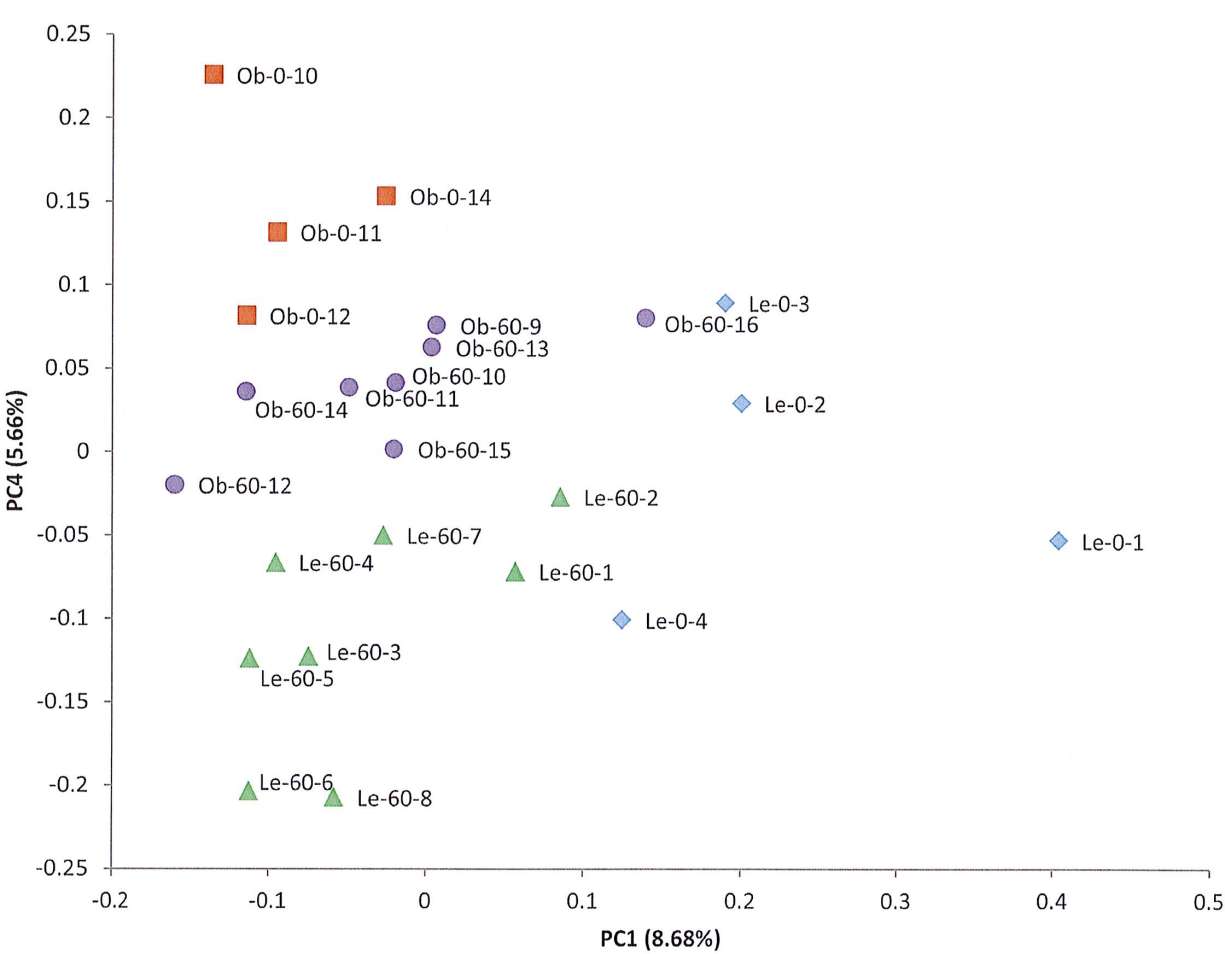
Principal coordinate analysis of the intestinal microbiota of lean and obese Zucker rats. This chart plots beta diversity values generated by unweighted unifrac analysis as part of the *jackknifed_beta_diversity*.*py* workflow script in QIIME. Samples were rarefied to 124,000 sequences per sample. Sample groups are indicated by symbol shape and color, and symbols are labeled with the individual sample names.

Table 1 contains the calculated alpha diversity of the bacterial populations within each sample as expressed through the Shannon Diversity, Chao1, Observed OTUs, and PD Whole Tree indices. As a group, the greatest calculated diversity was found in the day 0 lean rats with an average Shannon index of 7.12 ± 0.52 while the lowest diversity was observed in the day 60 lean rats (Shannon index 6.33± 0.35). Statistical analysis indicated that there were no significant differences in the alpha diversity of the bacterial populations in the different test groups (S3 Fig). Principal Coordinate Analysis (PCoA) of the bacterial populations from each animal showed clear clustering of the samples within each test group, suggesting a greater similarity of the populations within each group than between the groups (Fig 4).

## Discussion

Zucker rats (*fa/fa*) are widely used as rat model to investigate the effects of early onset of obesity on the development of several different diseases [25]. Obesity in the Zucker rat is inherited as an autosomal recessive trait caused by a mutation in the leptin receptor gene (fa). Animals homozygous for the *fa* allele become noticeably obese by 3 to 5 weeks of age, and by 14 weeks of age, their body composition is more than 40% lipid. The most valuable contribution of the Zucker rat has been its utility as a model of human early onset of obesity. Multiple researchers have used the obese Zucker rat model to investigate the cause and possible treatment of obesity related diseases [25–35]. The lean and obese Zucker rats used in the study were generated by breeding a heterozygous female and heterozygous male, so the lean and obese rats come from the same colony; however the lean and obese rats were not siblings. Understanding the relationship between obesity, the intestinal microbiota and the potential impact on disease in lean and obese Zucker rats is important due to their widespread use a model disease system. This study begins to address obesity and its impact on the microbiome in these animals, which lays the foundation for future studies incorporating disease states.

To examine the differences in the microbial populations of obese and lean rats at different ages, this study used next generation DNA sequencing of 16S rRNA genes present in DNA extracted from fecal samples. This approach generated large numbers of sequences per sample and allows for the identification and enumeration of less prevalent constituents of the gut bacterial population.

Multiple studies have presented analysis of the intestinal bacteria of lean and obese subjects, including several different laboratory animals and human subjects. Many of these studies have associated differences in the Firmicutes to Bacteroidetes ratios to the lean or obese state. Obese mice showed higher levels of Firmicutes and correspondingly lower levels of Bacteroidetes than their lean counterparts, whether the obesity was genetically predisposed [5] or diet-induced [17]. Similar differences in Firmicutes/Bacteroidetes ratios have been observed in rat [36], pig [37], and human [7] obesity studies. For the Zucker rats in this study, on day 0 the lean rats had much higher levels of Bacteroidetes (and lower Firmicutes/Bacteroidetes ratios) than the obese rats, but by day 60, the ratios were similar for both lean and obese animals. These results contrast with a simplistic association of high Firmicutes/Bacteroidetes ratios with obesity that has been often reported. Other work has also reported no clear association of the Firmicutes/Bacteroidetes ratios and obesity and that the intestinal microbiota of obese individuals does not have a simple taxonomic signature [38].

There have been few previous studies that have examined the microbiome in Zucker rats, and these studies were carried out using somewhat different approaches. Lees et al. [39] examined the microbial communities of 6 sets of fa/fa obese, fa/+ lean, and +/+ lean male Zucker rats that were housed together (one of each strain in each set) for 10 weeks in cages rather than the single cages, as we used in this study. Their findings indicated that rats housed together had similar fecal microbial populations to one another, regardless of obesity status. These findings are not overly surprising in that rats tend to be coprophagic and thus the ingestion of each other’s feces likely leads to a confluence of the microbial populations among cage mates. The study also found changes in the microbial populations at different ages, which is similar to our findings, especially with the lean rats. Similar to the present study, there was an increase in Rumincoccaceae among both lean and obese rats, an increase in Bifidobacteriaceae (seen in obese rats in the current study), and a reduction of Bacteroidaceae (seen in the lean rats in the current study) as the rats aged. Cases where we only saw the changes in either the lean or obese animals in contrast to Lees et al. [39] may be due to the influence of housing the animals separately, thereby reducing the potential for cross colonization that likely led to common changes across the different obesity classes of the rats.

A second study on the intestinal microbiota conducted by Gu et al. [36] examined the bacterial populations from samples collected from different gastrointestinal tract locations (stomach, duodenum, jejunum, ileum, cecum and feces) at a single time point (14 weeks of age) from 3 fa/fa obese and 3 lean male Zucker diabetic fatty (ZDF) rats [36]. The ZDF rats are a model of type 2 diabetes, distinct from the lean and obese rats used in the current study, in that they will become obese and exhibit insulin resistance. The findings from the fecal microbiota were different between the two studies in that there was a much higher proportion of Bacteroidetes in the rats from the Gu et al. [36] study compared to the current study (~55% vs. ~13–38%, respectively). The microbial compositions in the current study were much closer to those detected in the samples collected from the ceca in the previous study. The variability in the fecal compositions may be due to dietary differences between the two studies or differences in the DNA extraction methods [40, 41]. Both this study and Gu et al. used the same target regions for Illumina-based sequencing of the 16S rRNA genes for population characterization, so this was not likely a source of variation. Thus relative to the other Zucker rat microbiota studies, the results of the current study are important because they suggest that observed microbiota differences are more associated with variation in host genetics than housing situation.

Our study also identified some lower abundance taxa that were not readily reported in the previous studies. The ability to detect these taxa may be due to the high number of sequencing reads per sample that averaged 291,531 in our study compared to 24,016 [36] and 536 [39] in the comparison studies. The high coverage allowed for some additional analyses of lower abundance taxa. For example, *Akkermansia muciniphila* was detected at higher percentage in the obese compared to the lean animals regardless of age. *A*. *muciniphila* is known to be a mucin degrading bacteria that has been suggested to play a role in gastrointestinal tract health and glucose stabilization [42]. Studies have also indicated that in mice, the species is associated with lean animals as compared to those with diet induced obesity [43,44]. These findings are seemingly at opposition to the findings in the current study, where obese rats have higher proportions of *Akkermansia* than most of the lean animals. Some major differences in the studies are the animal model species (mice vs. rats) and basis of obesity (dietary vs. genetic), which could play a role in the difference in findings. In order to induce obesity, many of the studies relied upon a high-fat diet for the obese animals vs. a lower fat diet for control animals [43,45,46] compared to the current study where the obese and lean animals were fed the same diets. Thus taken together, the relationship between diet, obesity, and *A*. *muciniphila*, warrants further investigation in the Zucker rat model.

In summary, we found that there are differences between gut microbiota of the lean and obese rats using the Zucker rat model. Since obesity is linked to the risk of development of different chronic diseases, this animal model would be valuable in the investigation of the interrelationship of obesity and the intestinal microbiota on the development of several human obesity-related diseases such as liver disease, cardiovascular disease, kidney disease, diabetes, and certain types of cancers. The benefit of using this obese model is that rats become obese in a short period of time which provides a better translation to obese humans. Consequently, this study provides baseline data on the interaction of obesity and gut microbiota in the Zucker rat model, which can be used for future targeted investigations of obesity, gut microbiota and several related diseases.

## Supporting information

S1 Fig Alpha rarefaction of microbiota populations from lean and obese Zucker rats. This graph shows alpha rarefaction curves from the microbiome analysis of the individual Zucker rat fecal samples. Rarefaction data were generated using the *alpha_rarefaction*.*py* script from QIIME, and values from the PD Whole Tree analysis are plotted.
(PDF)

S2 Fig LEfSe analysis of microbiota differences for lean and obese Zucker rats at days 0 and 60. The histogram shows the Linear Discriminant Analysis (LDA) scores for bacterial classifications that are significantly elevated in the Lean_0 (red), Lean_60 (green), Obese_0 (blue) or Obese_60 (purple) groups. Bars are labeled with the most stringent bacterial classification. Letters to the right of the bars correspond to the letters in the key of the circular cladogram. The cladogram displays the phylogenetic relationship of the bacterial classifications determined to be statistically distinct between the four sample groups.
(PDF)

S3 Fig Statistical analysis of alpha diversity. Parametric t-test analysis of the alpha diversity of the test groups was performed using the *compare_alpha_diversity*.*py* script from QIIME at a rarefaction depth of 124,000 sequences per sample. Panel A contains the pairwise comparison of the different test groups using Chao1, Observed OTUs, and PD Whole Tree metrics. Data presented includes mean values, standard deviations, t statistics, and p values for each group comparison. Panels B, C, and D show boxplots of the Chao1, Observed OTUs, and PD Whole Tree alpha diversity statistics, respectively. Plus (+) symbols on the boxplots represent individual samples that fall outside the calculated distribution.
(PDF)

S1 Table QIIME mapping file. (PDF)

S2 Table Classification of the lean and obese Zucker rat intestinal microbiomes. This table shows the classification of the OTUs based on QIIME analysis using the Greengenes database at the Family and Genus level. Values in the table represent the fraction of the total OTUs within each sample that matched the taxonomic identification.
(CSV)

S3 Table Population changes in the predominant families of the order Bacteroidales from day 0 to day 60. (PDF)
