# Supplementary figures and images for: Assessment of gut microbiota populations in lean and obese Zucker rats
Source: PLoS One. 2017 Jul 13;12(7):e0181451. doi: 10.1371/journal.pone.0181451 (PMC5509373; doi:10.1371/journal.pone.0181451)

S1 Fig. Alpha rarefaction of microbiota populations from lean and obese Zucker rats

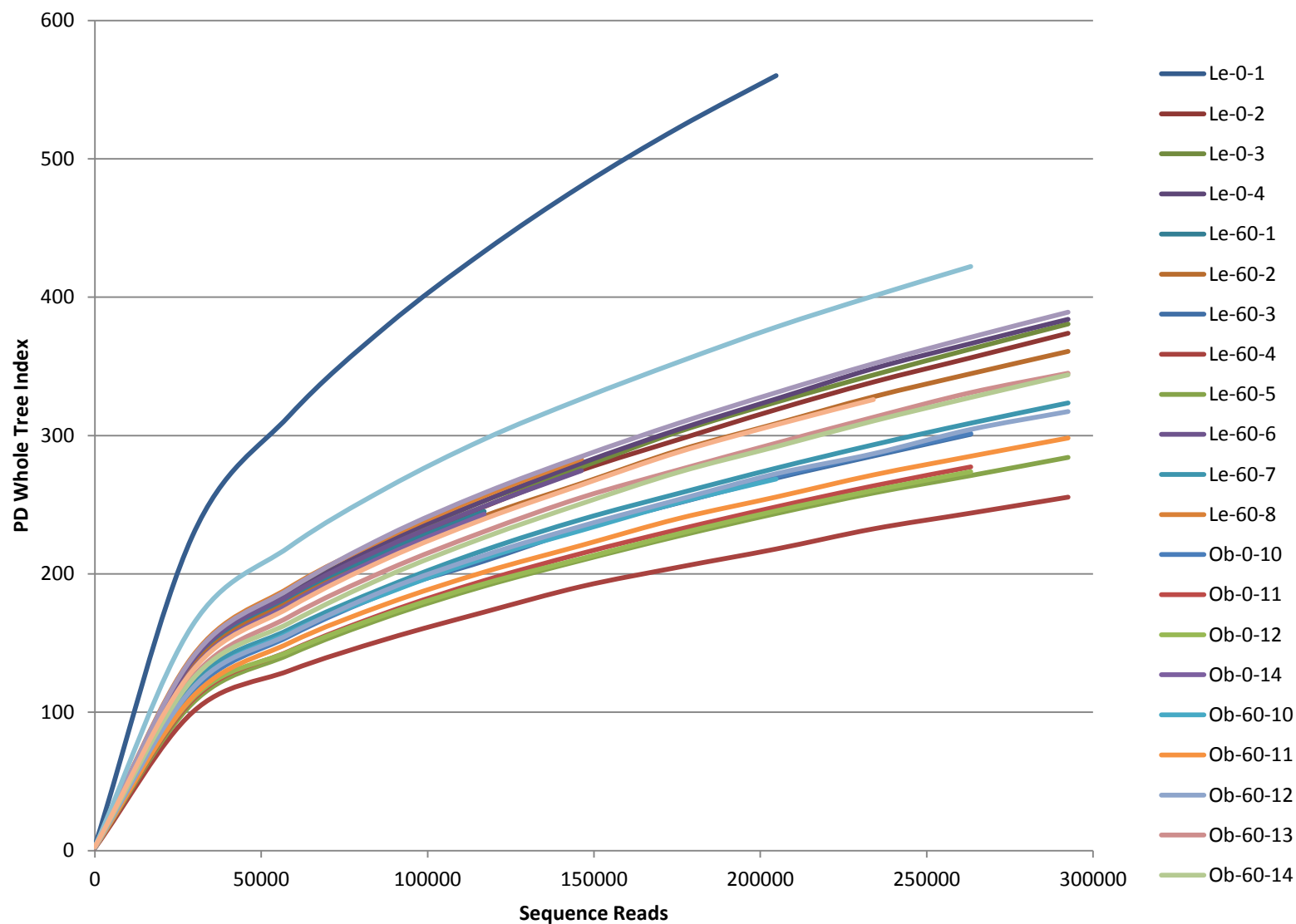

Supplement: S1 Fig — This graph shows alpha rarefaction curves from the microbiome analysis of the individual Zucker rat fecal samples. Rarefaction data were generated using the alpha_rarefaction.py script from QIIME, and values from the PD Whole Tree analysis are plotted. (PDF) [file pone.0181451.s001.pdf]

Lean\_0 Lean\_60 Obese\_0 Obese\_60

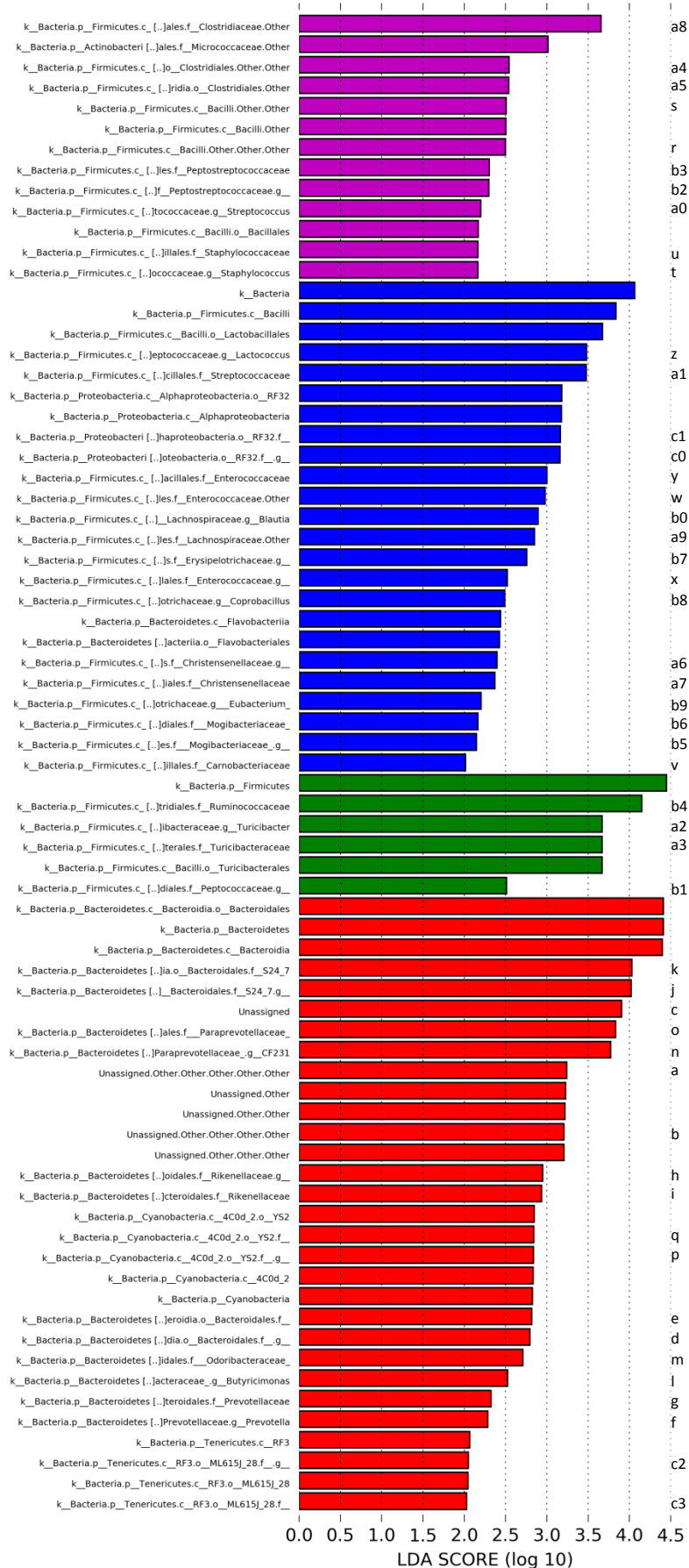

Supplement: S2 Fig — The histogram shows the Linear Discriminant Analysis (LDA) scores for bacterial classifications that are significantly elevated in the Lean_0 (red), Lean_60 (green), Obese_0 (blue) or Obese_60 (purple) groups. Bars are labeled with the most stringent bacterial classification. Letters to the right of the bars correspond to the letters in the key of the circular cladogram. The cladogram displays the phylogenetic relationship of the bacterial classifications determined to be statistically distinct between the four sample groups. (PDF) [file pone.0181451.s002.pdf]
