# Supplementary material for: Assessment of gut microbiota populations in lean and obese Zucker rats
Source: PLoS One. 2017 Jul 13;12(7):e0181451. doi: 10.1371/journal.pone.0181451 (PMC5509373; doi:10.1371/journal.pone.0181451)

S3 Fig. Statistical analysis of alpha diversity

A.

Chao1

Group1	Group2	Group1 mean	Group1 std	Group2 mean	Group2 std	t stat	p-value
Obese_60	Lean_60	13170.0679	1786.706341	12777.56854	1951.859171	0.39244124	1
Lean_60	Obese_0	12777.56854	1951.859171	11741.73367	1658.27603	0.830554886	1
Lean_0	Lean_60	19137.73889	6537.864647	12777.56854	1951.859171	2.314016948	0.259265015
Obese_60	Obese_0	13170.0679	1786.706341	11741.73367	1658.27603	1.220229125	1
Obese_60	Lean_0	13170.0679	1786.706341	19137.73889	6537.864647	-2.198332169	0.315535833
Lean_0	Obese_0	19137.73889	6537.864647	11741.73367	1658.27603	1.899253371	0.637678747

Observed_OTUs

Group1	Group2	Group1 mean	Group1 std	Group2 mean	Group2 std	t stat	p-value
Obese_60	Lean_60	4600.35	693.3376414	4447.4125	764.3299622	0.39210741	1
Lean_60	Obese_0	4447.4125	764.3299622	3950.375	522.0880499	1.069091989	1
Lean_0	Lean_60	6349.2	2264.089085	4447.4125	764.3299622	1.957202407	0.472807512
Obese_60	Obese_0	4600.35	693.3376414	3950.375	522.0880499	1.51074674	0.970727125
Obese_60	Lean_0	4600.35	693.3376414	6349.2	2264.089085	-1.830148153	0.582913426
Lean_0	Obese_0	6349.2	2264.089085	3950.375	522.0880499	1.788197935	0.743770701

PD_whole_tree

Group1	Group2	Group1 mean	Group1 std	Group2 mean	Group2 std	t stat	p-value
Obese_60	Lean_60	241.2600974	29.65623007	228.7073553	29.09298504	0.799430287	1
Lean_60	Obese_0	228.7073553	29.09298504	215.7256385	20.87078857	0.726541708	1
Lean_0	Lean_60	304.574262	80.9333791	228.7073553	29.09298504	2.157564024	0.338044395
Obese_60	Obese_0	241.2600974	29.65623007	215.7256385	20.87078857	1.407362375	1
Obese_60	Lean_0	241.2600974	29.65623007	304.574262	80.9333791	-1.793394174	0.618934718
Lean_0	Obese_0	304.574262	80.9333791	215.7256385	20.87078857	1.841209493	0.691109538

B.

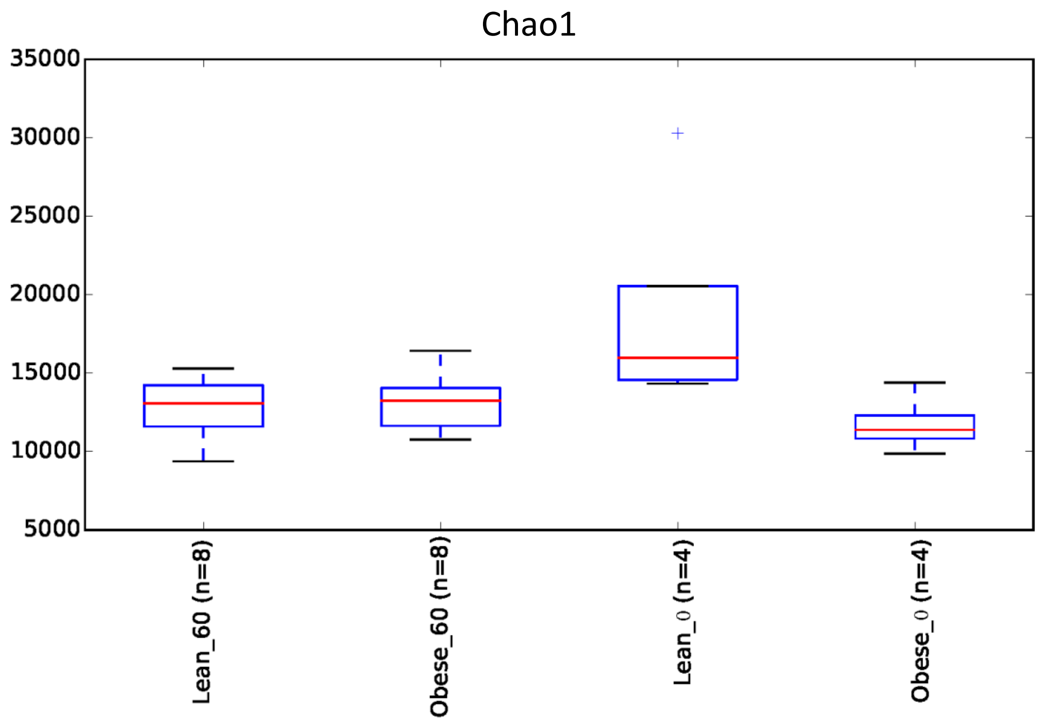

S3 Fig. Statistical analysis of alpha diversity among the test groups

C.

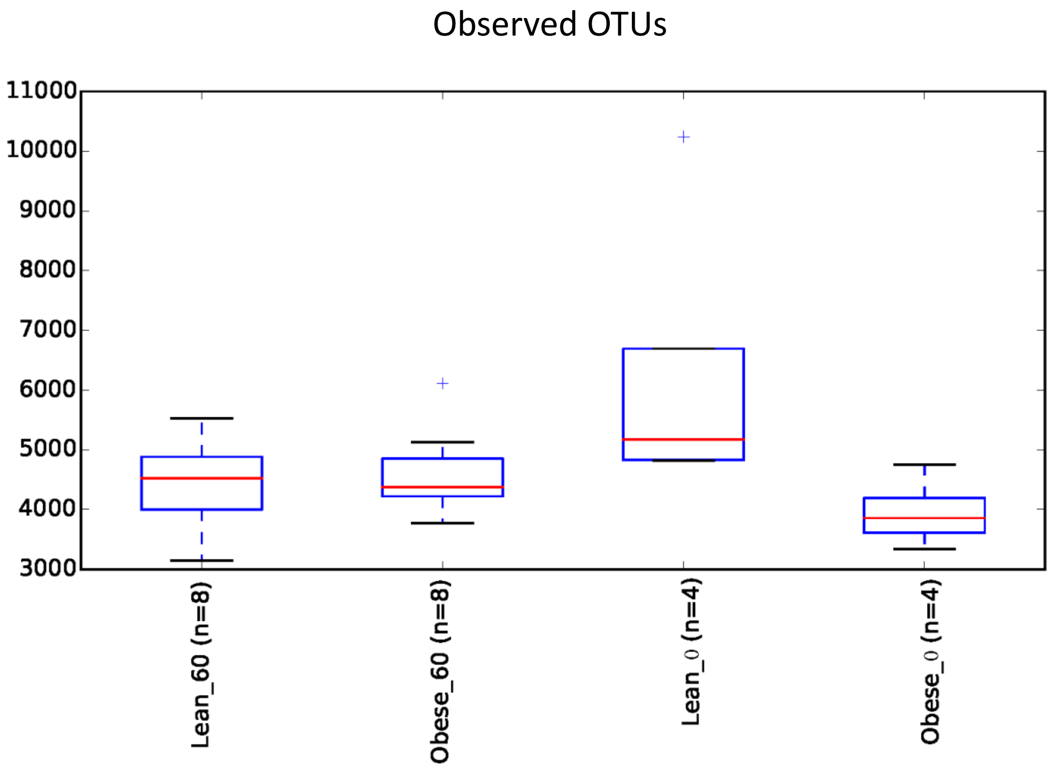

D.

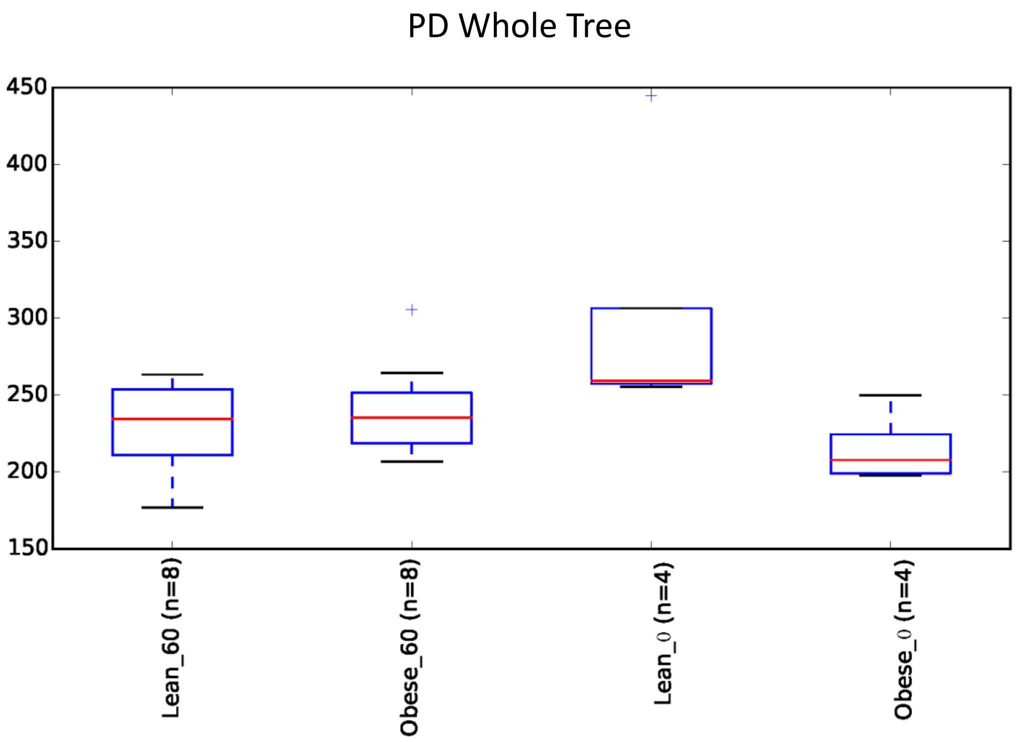

Supplement: S3 Fig — Parametric t-test analysis of the alpha diversity of the test groups was performed using the compare_alpha_diversity.py script from QIIME at a rarefaction depth of 124,000 sequences per sample. Panel A contains the pairwise comparison of the different test groups using Chao1, Observed OTUs, and PD Whole Tree metrics. Data presented includes mean values, standard deviations, t statistics, and p values for each group comparison. Panels B, C, and D show boxplots of the Chao1, Observed OTUs, and PD Whole Tree alpha diversity statistics, respectively. Plus (+) symbols on the boxplots represent individual samples that fall outside the calculated distribution. (PDF) [file pone.0181451.s003.pdf]
