# Supplementary material for: Assessment of gut microbiota populations in lean and obese Zucker rats
Source: PLoS One. 2017 Jul 13;12(7):e0181451. doi: 10.1371/journal.pone.0181451 (PMC5509373; doi:10.1371/journal.pone.0181451)
Supplement: S1 Table — (PDF) [file pone.0181451.s004.pdf]

S1 Table. QIIME mapping file

#SampleID	BarcodeSequence	LinkerPrimerSequence	ReversePrimer	Group	Description
le.60.1	ACAACTACT	CCTACGGGNGGCWGCAG	GACTACHVGGGTATCTAATCC	Lean_60	lean_day_60_rat_1
le.60.2	CACGGAACT	CCTACGGGNGGCWGCAG	GACTACHVGGGTATCTAATCC	Lean_60	lean_day_60_rat_2
le.60.3	GCCGAAACT	CCTACGGGNGGCWGCAG	GACTACHVGGGTATCTAATCC	Lean_60	lean_day_60_rat_3
le.60.4	ATTCGTACT	CCTACGGGNGGCWGCAG	GACTACHVGGGTATCTAATCC	Lean_60	lean_day_60_rat_4
le.60.5	AGGAATACT	CCTACGGGNGGCWGCAG	GACTACHVGGGTATCTAATCC	Lean_60	lean_day_60_rat_5
le.60.6	CGGCCAACT	CCTACGGGNGGCWGCAG	GACTACHVGGGTATCTAATCC	Lean_60	lean_day_60_rat_6
le.60.7	ATGGTAACT	CCTACGGGNGGCWGCAG	GACTACHVGGGTATCTAATCC	Lean_60	lean_day_60_rat_7
le.60.8	CTCCGTACT	CCTACGGGNGGCWGCAG	GACTACHVGGGTATCTAATCC	Lean_60	lean_day_60_rat_8
ob.60.9	ACCTGGACT	CCTACGGGNGGCWGCAG	GACTACHVGGGTATCTAATCC	Obese_60	obese_day_60_rat_9
ob.60.10	CGAAGTACT	CCTACGGGNGGCWGCAG	GACTACHVGGGTATCTAATCC	Obese_60	obese_day_60_rat_10
ob.60.11	TCGTGGACT	CCTACGGGNGGCWGCAG	GACTACHVGGGTATCTAATCC	Obese_60	obese_day_60_rat_11
ob.60.12	ACACAAACT	CCTACGGGNGGCWGCAG	GACTACHVGGGTATCTAATCC	Obese_60	obese_day_60_rat_12
ob.60.13	TCTTGAACT	CCTACGGGNGGCWGCAG	GACTACHVGGGTATCTAATCC	Obese_60	obese_day_60_rat_13
ob.60.14	CCAAGTACT	CCTACGGGNGGCWGCAG	GACTACHVGGGTATCTAATCC	Obese_60	obese_day_60_rat_14
ob.60.15	TCGCGAACT	CCTACGGGNGGCWGCAG	GACTACHVGGGTATCTAATCC	Obese_60	obese_day_60_rat_15
ob.60.16	CGGTCGACT	CCTACGGGNGGCWGCAG	GACTACHVGGGTATCTAATCC	Obese_60	obese_day_60_rat_16
le.0.1	TATCGGACT	CCTACGGGNGGCWGCAG	GACTACHVGGGTATCTAATCC	Lean_0	lean_day_0_rat_1
le.0.2	AGAAGAACT	CCTACGGGNGGCWGCAG	GACTACHVGGGTATCTAATCC	Lean_0	lean_day_0_rat_2
le.0.3	ACTGTTACT	CCTACGGGNGGCWGCAG	GACTACHVGGGTATCTAATCC	Lean_0	lean_day_0_rat_3
le.0.4	TCTCAAACT	CCTACGGGNGGCWGCAG	GACTACHVGGGTATCTAATCC	Lean_0	lean_day_0_rat_4
ob.0.10	TTCCTTACT	CCTACGGGNGGCWGCAG	GACTACHVGGGTATCTAATCC	Obese_0	obese_day_0_rat_10
ob.0.11	TTCGTAACT	CCTACGGGNGGCWGCAG	GACTACHVGGGTATCTAATCC	Obese_0	obese_day_0_rat_11
ob.0.12	CTTCCGACT	CCTACGGGNGGCWGCAG	GACTACHVGGGTATCTAATCC	Obese_0	obese_day_0_rat_12
ob.0.14	GTCCGTACT	CCTACGGGNGGCWGCAG	GACTACHVGGGTATCTAATCC	Obese_0	obese_day_0_rat_14
