# Supplementary material for: Assessment of gut microbiota populations in lean and obese Zucker rats
Source: PLoS One. 2017 Jul 13;12(7):e0181451. doi: 10.1371/journal.pone.0181451 (PMC5509373; doi:10.1371/journal.pone.0181451)
Supplement: S3 Table — (PDF) [file pone.0181451.s006.pdf]

Predominant families of the order Bacteroidales

	Average % of total microbiota				% change from day 0 to 60	
	Lean-0	Obese-0	Lean-60	Obese-60	Lean	Obese
Bacteroidaceae	11.53	9.15	6.70	8.65	-42	-5.5
S24-7	15.76	5.62	4.57	4.98	-71	-11
Paraprevotellaceae	7.08	0.93	0.34	0.66	-95	-29
